# Standardized epidemiological protocols for populations affected by volcanic eruptions

**DOI:** 10.2471/BLT.19.244509

**Published:** 2020-03-02

**Authors:** William Mueller, Hilary Cowie, Claire J Horwell, Peter J Baxter, Damien McElvenny, Mark Booth, John W Cherrie, Paul Cullinan, Deborah Jarvis, Ciro Ugarte, Hiromasa Inoue

**Affiliations:** a Institute of Occupational Medicine, Research Ave N, Currie, EH14 4AP, Scotland.; b Department of Earth Sciences, Durham University, Durham, England.; c Department of Public Health and Primary Care, University of Cambridge, Cambridge, England.; d Faculty of Medical Sciences, Newcastle University, Newcastle upon Tyne, England.; e Faculty of Medicine, Imperial College London, London, England.; f Pan American Health Organization, Washington, DC, United States of America.; g Department of Pulmonary Medicine, Kagoshima University, Kagoshima, Japan.; Correspondence to William Mueller (email: will.mueller@iom-world.org).

Volcanic eruptions can have far-reaching consequences for human health, including injuries, illnesses and deaths. One study estimates that over 270 000 people lost their lives in volcanic episodes between 1600 and 2010, with 600 million people currently living in areas of risk.^1^ Following an eruption, air quality can be significantly deteriorated. Since airborne ash and gases from volcanic emissions may affect the respiratory system,^2^ the public may be concerned over the potential health effects, particularly those most exposed or most vulnerable.^3^ Studies of the health impacts associated with volcanic hazards started with the eruption of Mount St. Helens, United States of America, in 1980. Since then, studies, mainly conducted in high-income countries, have identified mostly reversible, short-term respiratory outcomes, with few studies undertaken for chronic outcomes.^4^

A key challenge to identifying and quantifying health impacts from volcanic eruptions has been the lack of consistent epidemiological protocols that can be rapidly deployed at eruption onset, which is an issue across disaster epidemiology.^5^ Conducting health research in settings of volcanic eruptions is inherently difficult because it involves disaster zones with highly mobile populations and temporary shelters.^6^ The lack of consistent data means that we still have a relatively poor understanding of the extent of health impacts from exposure to volcanic emissions, due to the challenges of interpreting studies of variable designs and robustness.

To address these difficulties and assist with the generation of health evidence from volcanic contexts, the International Volcanic Health Hazard Network has developed standardized protocols to facilitate epidemiological studies of populations that have been, or may be in the future, exposed to volcanic ash and gases. We describe here the process, content and suggested use of these protocols to help promote awareness and build the health evidence base of acute volcanic exposures. These protocols are available for free download on the network’s website.^7^ Although we focus on volcanic eruptions, all acute exacerbations of air quality, such as from wildfires, warrant study, which may be facilitated by adapting the network’s protocols to other such exposure scenarios.

## Methods and process

As part of the network’s Health Interventions in Volcanic Eruptions research project, we developed protocols to enable two types of studies: (i) a basic study tallying hospital and clinic visits of respiratory (and potentially other) health outcomes, to be conducted during and/or immediately following a volcanic eruption, where syndromic surveillance is not already in place; and (ii) a more detailed, cross-sectional survey of individuals exposed to volcanic emissions, which may be undertaken if the basic study or syndromic surveillance indicates adverse health effects.

The protocols were subject to several rounds of internal review by a panel consisting of expert researchers and advisory group members from Japan, the United Kingdom of Great Britain and Northern Ireland and the USA, resulting in final draft versions for wider circulation. The next stage of protocol development was to seek peer review from other experts in epidemiological research and with individuals and agencies who would be responsible for implementing the protocols in the field. This collaborative approach was essential to ensure that the final protocols were fit for purpose, including incorporation of logistical, economic and cultural factors that may influence study implementation, and contained all of the information necessary for their application, at short notice, in potential emergency situations. An important consideration at this stage was the harmonization of the protocols with other emergency response activities to minimize any real or perceived burden from the added efforts.

Two workshops were held to develop the protocols. The first focused on the technical aspects and content of the protocols, ensuring that they were comprehensive and jargon-free. The second, to present and discuss the protocols, took place after peer review and assessing issues of practical implementation. The workshop was hosted by the Pan American Health Organization, and delegates included representatives from implementing agencies from Argentina, Chile, Costa Rica, Ecuador, Nicaragua, Peru and St Vincent and the Grenadines. Final versions of the protocols were produced following incorporation of feedback from this workshop.

## Outputs and uses

### The protocols

The basic study offers a simple design allowing a quick survey at the population level to identify any increase in specific morbidity indicators in the areas exposed to volcanic emissions, compared to the period before the volcanic event or to a similar, but unexposed area.^8^ The cross-sectional study examines ill health and estimated exposures to volcanic emissions at an individual level, with the aim of determining whether health effects occur more commonly in those areas with higher exposures.^9^ A cross-sectional approach takes advantage of individual-level exposure and health data, rather than at an ecological level, thus providing higher confidence in study results.

While other study designs are possible, including case-control and longitudinal designs, these approaches typically involve more nuanced methods (for example, selecting controls) and higher costs.^10^ Case-control studies are often employed when the disease under investigation is rare and would not be needed for relatively common exacerbations and symptoms of respiratory illness.^11^ Longitudinal studies typically take longer and are resource-intensive, so are not suitable for shorter-term increases in adverse health effects. Nevertheless, the basic and cross-sectional study designs we present entail their own challenges. For example, the size of the population at risk before and after the eruption (that is, the denominator in a basic study) should be quantified, yet this may be hindered by the degree of population movement following a volcanic episode. Similarly, risks identified by cross-sectional studies might be underestimated in chronically exposed areas, where more vulnerable subgroups may be less likely to reside. Ultimately, such issues might be unavoidable, but are necessary to consider when interpreting study results.

Both protocols include guidance on the key aspects of study design, including size and location of study; study population and recruitment; exposure assessment (including ambient air quality monitoring/sampling, timeframes and geocoding of addresses); health outcomes; data collection (including health and demographic data); and data analysis and interpretation.

### Use of the protocols

The intention of these protocols is to be applicable in all volcanic contexts and settings, regardless of resource availability, health records systems or magnitude and duration of eruption. The overarching purpose of the protocols is to determine whether a short-term increase in adverse health outcomes following a volcanic eruption, including, for example, injuries, burns or respiratory outcomes, exists.

Compared to other natural disasters, there is potentially a wide range of causes of death, injury and other health impacts corresponding to the multiple phenomena of eruptions and volcanic behaviour, which vary both among eruptions and volcanoes.^2^ The studies, therefore, have to be undertaken in close collaboration with the volcanologists who advise the authorities on the hazards that may arise in a particular crisis, both before and after eruptive events.

The basic study protocol can be of use to governmental and relevant health agencies, research institutes and hospitals that wish to assess in a timely manner the respiratory and other potential health effects in exposed populations. The focus in developing this standardized protocol was on efficiency, cost containment and on providing information to the public on the health risks at the earliest opportunity. While undertaking this study in a disaster setting will be difficult amidst the many other emergency management responsibilities, the data collection forms included in the protocol can be integrated into response activities for efficiency and to minimize any duplication. Efforts to undertake a study during or following an eruption should be coordinated with concurrent emergency responses or other associated activities.

The cross-sectional protocol can be of use to governmental and nongovernmental health agencies or research institutes that wish to produce more detailed information than that provided by the basic study, to assess the extent to which exposure to volcanic emissions is associated with adverse respiratory and other health effects. The cross-sectional study requires more resources and time than the basic study but, as a result, generates more detailed data and creates the possibility of continued research. Study findings can be used to encourage people to reduce their exposures to ash and hazardous gases whenever possible, and can also provide useful baseline data for continued follow-up of the same population. Such longitudinal cohort studies would provide valuable long-term health evidence, which is currently very limited.

In addition to more extensive data collection, numerous other required activities should be considered when developing timelines and budgeting, including planning, training, submitting and receiving study clearances (for example ethical clearances), recruitment, data analysis and disseminating results. Whereas the basic protocol could potentially be completed within the weeks following an eruption, the cross-sectional study would likely require months before results would be available.

Health impacts ought to be at the forefront of volcanic risk assessments, decision-making on timely evacuations of populations and on the provision of evidence-based public health messaging to vulnerable groups exposed to volcanic emissions. By advising relatively simple and inexpensive methods, these protocols should provide the necessary first steps in supporting surveillance and tracking early health signals from inhalation of volcanic emissions. As more data sources become readily available, for example satellite imagery, social media, mobile data and electronic records, surveillance and research tools to detect and integrate exposure, mobility and health endpoints will likely be enhanced.^12^ Furthermore, if these protocols are implemented across different geographic settings, there may be an opportunity to pool results to reach even stronger conclusions about the potential health effects from exposure to volcanic emissions.
